# *Liparis aphylla* (Malaxideae, Orchidaceae), a new leafless record from Peru

**DOI:** 10.3897/phytokeys.61.7420

**Published:** 2016-02-25

**Authors:** Alexander Damian, Paul Ormerod

**Affiliations:** 1 Facultad de Ciencias Ambientales. Universidad Científica del Sur. Panamericana Sur Km. 19, Lima, Peru; 2 P.O. Box 8210, Cairns 4870, Queensland, Australia

**Keywords:** Orchidaceae, *Liparis aphylla*, Cajamarca, Peru, *Liparis* section *Aphylla*, Orchidaceae, Liparis aphylla, Cajamarca, Peru, *Liparis* section *Aphylla*

## Abstract

*Liparis aphylla* G.A.Romero & Garay was previously known only from two herbarium specimens collected in 1945 and 1977 in Ecuador and Colombia, respectively. This little-known species is hereby reported for the first time for Peru. An updated description, line illustration, color photographs and distribution map of *Liparis aphylla*, as well as an identification key to the Peruvian species of *Liparis* are provided.

## Abstract (es)

*Liparis aphylla* G.A.Romero & Garay solo se conocía de dos especímenes de herbario colectados en 1945 y 1977 en Ecuador y Colombia, respectivamente. Esta especie poco conocida se registra por primera vez para Perú. Se presenta una descripción actualizada, ilustración de línea, fotografías a color y un mapa de distribución de *Liparis aphylla*, así como una clave de identificación para las especies peruanas de *Liparis*.

## Introduction

*Liparis* Rich. *s.l.* is a large cosmopolitan genus of about 480 species, reported in tropical Asia, Malesia, eastern Australia, the Pacific Islands (including Hawaii and Tahiti), Madagascar, Africa, subtropical and tropical Americas, temperate Europe, Asia and North America. The genus is composed of both terrestrial and epiphytic species; with small to prominent pseudobulbs; one to several (rarely none) conduplicate to plicate leaves, a terminal inflorescence of one to many; flowers usually resupinate, small or medium-sized, yellow, green, orange, or purple; the sepals are similar, although the lateral ones are often wider and shorter than the dorsal one; the labellum is firmly attached to a footless, often arched column; the incumbent anther bears four pollinia grouped in two pairs, and lacking caudicles or stipe but with minute viscidium. According to a recent molecular phylogenetic study (Cameron 2005), *Liparis* contains four major clades, two of which include primarily Asiatic-Malesian epiphytic taxa with narrow conduplicate leaves, and the other two consist mostly of terrestrial plants with worldwide distribution and broader, conduplicate to plicate leaves. However, further studies are needed to better understand its evolutionary history and to clarify its delimitation and position within the tribe Malaxideae.

Schlechter (1921), compiled the first account of Liparis in Peru and reported three species, namely *Liparis crispifolia* Rchb.f, *Liparis elegantula* Kraenzl, and *Liparis ramosa* Poepp. & Endl. Later, Schweinfurth (1959) listed eight species, adding *Liparis elata* Lindl. [= *Liparis nervosa* (Thunb. ex A. Murray) Lindl.], *Liparis laticuneata* C. Schweinf., *Liparis retusa* Fawc. & Rendle, and *Liparis vexillifera* (Lex.) Cogn. Bracko & Zarucchi (1993) added two unvouchered records, namely *Liparis luerii* Dodson [= *Liparis serpens* Garay] and *Liparis neuroglossa* Rchb.f. to the list of Peruvian taxa (see Ormerod 2012). As far as we know, *Liparis neuroglossa* is only known from the Bolivian type collection. Taking into account the previous records and four additional Peruvian species described by Ormerod (2012), currently we recognize the following 11 species for this country: *Liparis ecallosa* Ormerod, *Liparis elegantula*, *Liparis laticuneata*, *Liparis nervosa*, *Liparis ramosa*, *Liparis retusa*, *Liparis rusbyi* Rolfe, *Liparis schunkei* Ormerod, *Liparis serratiloba* Ormerod, *Liparis vargasii* Ormerod, and *Liparis vexillifera*.

During field exploration conducted by the senior author in the montane rainforest of Cajamarca, Peru, in 2014, a small terrestrial individual plant of *Liparis* was collected and subsequently identified as *Liparis aphylla*. Because of the scarcity of information about this rare orchid, we provide an updated description and line illustration, we illustrate it with color photos for the first time and we provide additional information regarding its ecology and morphological variation.

## Materials and methods

A live plant of *Liparis aphylla* was collected in May 2014 in Cajamarca, Peru (see detailed locality data under “Additional specimen examined” below). Specimen identification was made by comparing the plant with the original publication of the species in Garay and Romero (1999). A herbarium specimen was prepared, and two flowers were preserved in a solution consisting of 70% ethanol, 20% water, and 10% glycerol. An updated description was prepared based on all collections of *Liparis aphylla* available (either physical specimens or digital images).

## Taxonomy

### Liparis aphylla

Taxon classificationPlantaeAsparagalesOrchidaceae

G.A.Romero & Garay

Figs 1 , 2

Liparis aphylla G.A.Romero & Garay. Harvard Pap. Bot. 4(2): 483. 1999.

#### Type.

COLOMBIA. Boyacá: Sierra del Cocuy, 2800 m, “terrestre, entre musgos asociada con *Masdevallia* sp., aparentemente saprófita; tépalos blanco-verdosos, labelo púrpura lila” 20 July 1997, *M. Ospina Hernández 1487* (Holotype: AMES!).

#### Description.

Herb, 4–10 cm tall, terrestrial. Rhizome and roots not seen. Pseudobulb subglobose, 2.5–5 × 2 mm, enveloped by a basal foliaceous green sheath 1.5 cm long. Leaves not seen. Inflorescence racemose, erect, successively (up to 6) flowered (usually two are open at a time), 7.8 cm. long; peduncle slender, 4 cm long; rachis weakly flexuous, distichous, 3.8 cm long; floral bracts lanceolate. acute, green, 5–7 × 3 mm. Flowers widely opening, resupinate, fragrance not detected, sepals and petals greenish, labellum rosy brown with a darker median stripe, column greenish suffused with rosy brown, pollinia yellow. Green, 4-6 long ovary with clavate, narrowly winged pedicel. Dorsal sepal oblong-lanceolate, obtuse, erect, 1–veined, 3–5.5 × 0.9–1.5 mm. Lateral sepals obliquely oblong-ovate, obtuse, midvein low carinate, parallell to each other under the labellum, 1-veined, 3–5 × 0.6–2 mm. Petals linear, obtuse, reflexed, 1–veined, 2.6–5 × 0.5–0.6 mm. Labellum subquadrate, distal margin serrate-denticulate, medially with a thickened glossy stripe, 3–5 × 3.5–5 mm; callus bilobed, each side with an erect, subquadrate lobe between which there is a distally thick-walled elliptical cavity. Column semiterete, thick basally but slender above, arcuate on its distal half, apex with small triangular wings on each side, 3 mm long; pollinia four in two pairs, waxy, triangular; anther cap ovoid. Capsule and seeds not seen.

#### Ecology and distribution.

*Liparis aphylla* is found in the Andes of Colombia, Ecuador and Peru, within an elevation range of 2600–3300 m. The distribution of this species, based on herbarium records, appears to be highly disjunct (Figure 3). However, this extreme patchiness may be an artifact of limited collecting, and we suspect that *Liparis aphylla* likely occurs throughout the Andean range, at climatically suitable locations ranging from the Cordillera Oriental/East Andes in Colombia to the northern Andes of Peru. Plants of *Liparis aphylla* grow terrestrially among loose moss in wet, cold montane cloud forest with abundant bryophytes. Flowering period: May–July.

**Figure 1.**
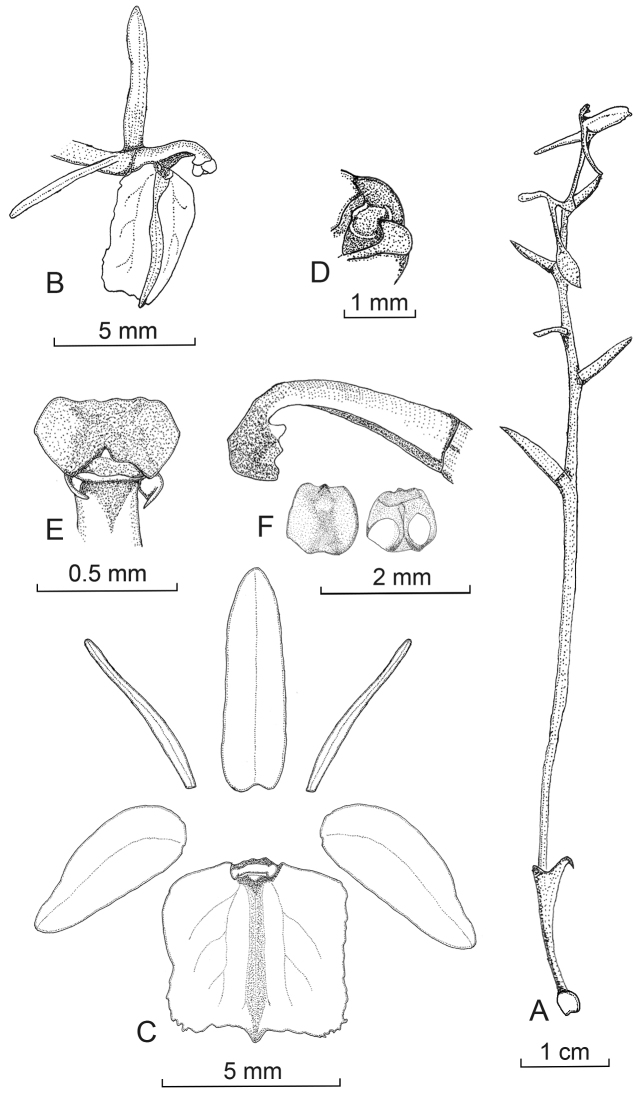
*Liparis aphylla*. **A** Habit **B** Flower **C** Dissected perianth **D** Detail of the labellum disk base **E** Column withouh the anter cap, ventral view **F** Column lateral view, and anther cap. Drawn from *A. Damian 0100* (MOL!).

**Figure 2.**
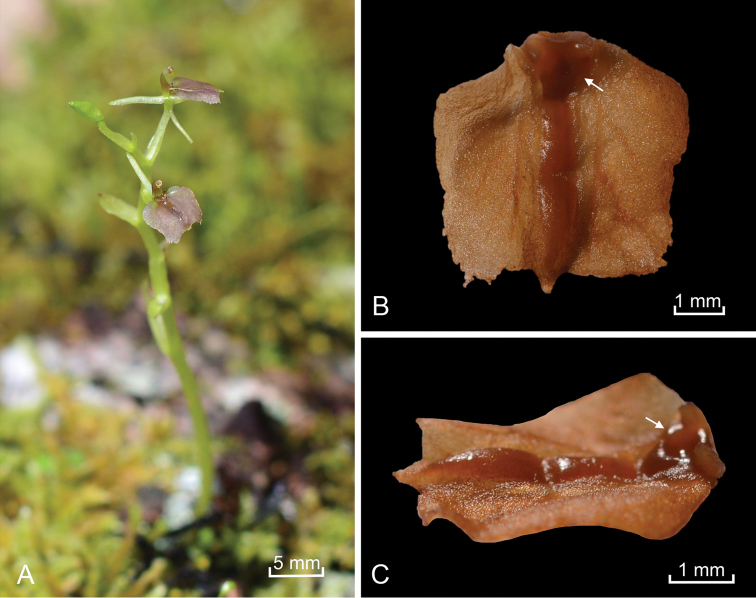
*Liparis aphylla*, **A** Habit **B–C** Two views of the of labellum. Arrows show the distinct elliptical concavity of the callus. Photographer: A. L. Damian.

**Figure 3.**
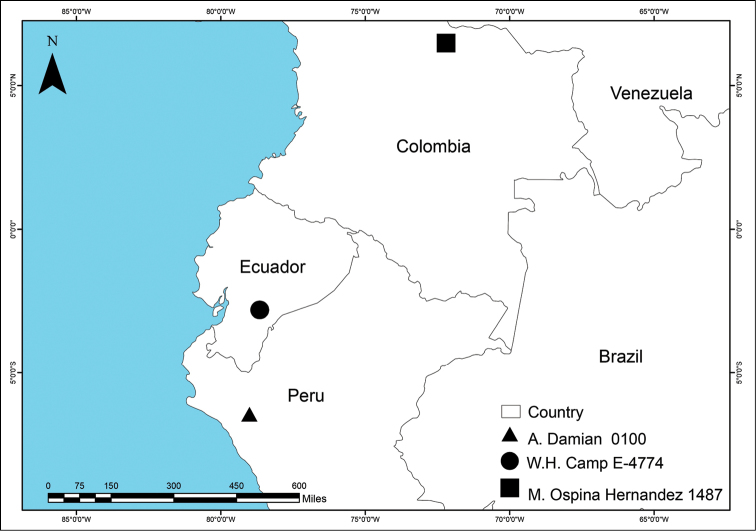
Known distribution of *Liparis aphylla*.

#### Additional specimens examined.

**ECUADOR.** Prov. Azuay: East Cordillera, 4–6 km N. of Sevilla de Oro, 2745–3050 m, 16 August 1945, W.H. Camp E-4774 (AMES!, NY); **PERU.** Departamento Cajamarca, provincia Chota, Querocoto, entrance road to “La Granja”, 6°20'6.70"S, 79°9'24.49"W; terrestrial, montane rainforest, 2600 m, 01 May 2014, *A. Damian 0100* (MOL!, ADP-spirit 3033).

#### Conservation status.

This species is presently know only from three location worldwide; according to the IUCN Red List (IUCN 2014) and Roque and Leon (2006) criteria, it should be listed as critically endangered (CR) B1ab(iii).

#### Discussion.

*Liparis aphylla* was described from an individual plant collected in Sierra del Cocuy, Colombia, by Romero and Garay (1997) and from another record from Azuay, Ecuador, (1977). These two specimens, along with the Peruvian specimen reported in this paper, represent the only available material of this tiny rare orchid. The overall morphology observed in these three specimens, is quite uniform except for considerable variation in labellum shape, which ranges from quadrate in the specimen *Camp E-4774* to subquadrate in *Ospina Hernández 1487*. Moreover, the elliptic concavity of the callus of our specimen did not appear to be present in other two specimens, although it is not clear if the absence of this concavity in the latter two specimens is an artifact of preservation.

Unlike any others members of *Liparis*, *Liparis aphylla* appears as a leafless orchid with poor-developed root system. However, these two conditions need to be studied carefully. Although we were unable to see any remnant roots or rhizome on the specimens examined, Ospina Hernandez sheet (1487) includes an interesting note cited as “Plant tubers covered by fungal hyphae (...)”. It is highly possible that “tubers” on this context actually refers to the pseudobulb and not to the presence of subterranean stems or shoots that resemble any kind of root-like system or rhizome as it occurs in many basal Epidendroids orchids (Pridgeon et al. 2005, Campbell 2014). A closest analysis of the original drawing of *Liparis aphylla* by Romero and Garay (1997) shows sort of filamentous structures emerging beneath the pseudobulb. Since the dimensions of these formations are indistinct (0.3 × 0.1 cm), is fairly accurate to attribute those filamentouse root-like structures to the “fungal hyphae” which Ospina was referring in the first place.

Another strikingly feature on *Liparis aphylla* is its leaflessness. As it happens with rhizome and roots, no remnants of withered or decomposed leaves were observed neither in the field nor in available herbarium specimens. As a result of this uncommon state within *Liparis*, Romero & Garay decided to establish *Sect. Aphylla* (Romero and Garay 1997) to include this single species, which outstands essentially for its leafless habit, well-developed pseudobulb, plants of small size and muscicolous habitat. However, additional observations whether this set of characters, especially those referring to leaves and roots, are continuous or not along specimens were missing.

Leaflessness is a feature that is present in many angiosperms (Vicent et al. 2013, Calswards et al. 2006) and Orchidaceae is not the exception. At least 235 orchid species and 43 genera are leafless, most of them found in Epidendroideae (Freudenstein and Barrett 2010). For instance, within tribe Malaxideae, two orchids have reported being leafless: *Malaxis aphylla* (King & Pantl.) T.Tang & F.T.Wang and *Malaxis saprophyta* (King & Pantl.) T.Tang & F.T.Wang (Vincent et al. 2013). Most of these leafless orchid display any of the following arrangements or life-forms: (1) well-developed shoot system which forms the main body (e.g. leafless *Vanilla*), (2) shoot system reduced, i.e. shootless orchids, roots forming the main body of the plant (e.g. Vandeae) (Carlsward et al. 2006), (3) roots fleshy, fasciculate, leaves basal but lacking at flowering time (e.g. Spiranthinae: Cranichideae) (Salazar 2003), and (4) myco-heterotrophic orchids, achlorophyllous, roots reduced or absent, rhizome fleshy, coralloid, tuberlike or cylindric (e.g. Aphyllorchis, Gastrodia) (Rasmussen 2000). A major question rise then among others, which life-form represents better to *Liparis aphylla*?. Although Romero and Garay (1997) suggested it could be referred to as a “saprophyte”, this term proved to be inaccurate (Leake 1994). We believed *Liparis aphylla* could represent a partially myco-heterotrophic (holo-mycotrophic) plant, i.e. clorophyllous plant that combines autotrophy and myco-heterotrophy to obtain carbon during at least one stage of its life cycle (Rasmussen 1995). The nonexistence of a well-developed root system, leaflessness (= myco-heterotrophic species) and retainment of chlorophyll on its basal sheath, stem and bracts seem to confirm this hypothesis. Nonetheless, it is important to keep in mind that the myco-heterotrophic status is “putative” on this species, and remains speculative until a careful physiological analysis has been carried out.

### Identification key to Peruvian species of *Liparis*

**Table:** 

1	Leaves absent	***Liparis aphylla***
–	Leaves present at flowering	**2**
2	Plants not decumbent; pseudobulbs or pseudobulb-like basal thickening present	**3**
–	Plants decumbent, pseudobulbs absent	**4**
3	Leaf solitary, basal or near the base	***Liparis vexillifera***
–	Leaves several, spreading, basal or near the base	***Liparis nervosa***
4	Leaves appearing singly on rhizome	**5**
–	Leaves appearing in pairs on rhizome or subapproximate on erect stem	**6**
5	Labellum subquadrate, ca. 7.5 mm wide	***Liparis retusa***
–	Labellum papilioforme, ca. 10.2 mm wide	***Liparis vargasii***
6	Labellum reniform	***Liparis serratiloba***
–	Labellum cuneate to pandurate or obovate	**7**
7	Labellum cuneate	**8**
–	Labellum elliptic, pandurate to obovate	**9**
8	Labellum disc with central thickened band	***Liparis laticuneata***
–	Labellum disc with minute bilobed forcipate callus	***Liparis elegantula***
9	Labellum lacking basal callus	***Liparis ecallosa***
–	Labellum with basal callus	**10**
10	Labellum elliptic-pandurate or narrowly obovate, to 6mm wide; basal callus V-shaped (edges apically convergent)	***Liparis schunkei***
–	Labellum suborbicular to broadly obovate, 9-12 mm wide; basal callus not V-shaped (edges parallel)	***Liparis rusbyi***

## Supplementary Material

XML Treatment for Liparis aphylla

## References

[B1] Brako L Zarucchi JL (1993) Catalogue of the flowering plants and Gymnosperms of Peru. Monographs in Systematic Botany from the Missouri Botanical Garden 45: 1–1286.

[B2] Cameron KM (2005) Leave it to the leaves: A molecular phylogenetic study of Malaxideae (Epidendroideae, Orchidaceae). American J. Bot. 92(6): 1025–1032. doi: 10.3732/ajb.92.6.1025 10.3732/ajb.92.6.1025 21652487

[B3] Campbell FK (2014) A summary of Holomycotrophic orchids. The MIOS Journal 15(4): 6–17.

[B4] Carlsward BS Whitten WM Williams NH Bytebier B (2006) Molecular Phylogenetics of Vandeae (Orchidaceae) and the evolution of leaflessness. Am. J. Bot. 93: 770–786. doi: 10.1007/978-1-4614-5209-6_2 21642140 10.3732/ajb.93.5.770

[B5] Freudenstein JV Barrett CF (2010) Mycoheterotrophy and diversity in Orchidaceae. In: Seberg O Petersen G Barfod AS Davis JI (Eds) Diversity, phylogeny, and evolution in the monocotyledons. Aarhus University Press, Arhus, 25–37.

[B6] Garay LA Romero-González GA (1999) Schedulae Orchidum II. Harvard Pap. Bot. 4(2): 475–488.

[B7] IUCN (2014) Guidelines for using the IUCN Red List categories and criteria. Version 8.1 Prepared by the Standards and Petitions Subcommittee in March 2014.

[B8] Leake JR (1994) The biology of Myco-heterotrophic (‘saprophytic’) plants. New Phytologist 127: 171–216. doi: 10.1111/j.1469-8137.1994.tb04272.x 10.1111/j.1469-8137.1994.tb04272.x 33874520

[B9] Ormerod P (2012) Notes on Liparis Section Ramosae (Orchidaceae: Malaxideae). Harvard Papers in Botany 17(1): 169–177. doi: 10.3100/025.017.0118

[B10] Pridgeon A Cribb PJ Chase MW Rasmussen FN (2005) Genera Orchidacearum, volume 4: Epidendroideae (Part One). Oxford University Press, Oxford, 1–608.

[B11] Rasmussen FN (2000) Ins and Outs of Orchid Phylogeny. In: Wilson KL Morrison DA (Eds) Monocots: Systematics and Evolution. CSIRO Melbourne, 430–435.

[B12] Rasmussen HN (1995) Terrestrial Orchids: From Seeds to Myco-trophic Plant. Cambridge University Press, Cambridge, 1–448. doi: 10.1017/cbo9780511525452

[B13] Roque J Leon B (2006) Orchidaceae endemicas del Peru. In: Leon B Pitman N Roque J (Eds) El libro rojo de las plantas endemicas el Peru. Rev. peru. biol. Numero especial 13(2): 759–878.

[B14] Salazar GA (2003) Spiranthinae. In: Pridgeon AM Cribb PJ Chase MW Rasmussen FN (Eds) Genera orchidacearum 3: orchidoideae part 2, Vanilloideae. Oxford University Press, Oxford, 164−278.

[B15] Schlechter R (1921) Die Orchideenflora der suderamikanischen Kordillerenstaaten IV. Peru. Rep. Sp. Nov. Regni Veg., Beih. 9: 1–182.

[B16] Schweinfurth C (1959) Orchids of Peru. Fieldiana, Bot. 30(2): 373–38.

[B17] Vincent SFT Frendenstein JV Kissling J Christenhusz JM Stotler ER Crandal-Stotler B Wickett N Rudall JP Maas-van de Kamer H Maas JM (2013) Taxonomy and Classiﬁcation. In: Vincent SFT (Ed.) Mycoheterotrophy: The Biology of Plants Living on Fungi. Springer Science+Business Media New York, 19–101.

